# Structural neighboring property for identifying protein-protein binding sites

**DOI:** 10.1186/1752-0509-9-S5-S3

**Published:** 2015-09-01

**Authors:** Fei Guo, Shuai Cheng Li, Zhexue Wei, Daming Zhu, Chao Shen, Lusheng Wang

**Affiliations:** 1 School of Computer Science and Technology, Tianjin University, 92 Weijin Road, Nankai District, Tianjin, People's Republic of China; 2 Department of Computer Science, City University of Hong Kong, 83 Tat Chee Avenue, Kowloon, Hong Kong; 3 School of Computer Science and Technology, Shandong University, 1500 Shunhua Road, Gaoxin District, Jinan, People's Republic of China

**Keywords:** Protein-Protein Binding Sites, Structural Neighboring Property, Voronoi Tessellation

## Abstract

**Background:**

The protein-protein interaction plays a key role in the control of many biological functions, such as drug design and functional analysis. Determination of binding sites is widely applied in molecular biology research. Therefore, many efficient methods have been developed for identifying binding sites. In this paper, we calculate structural neighboring property through Voronoi diagram. Using 6,438 complexes, we study local biases of structural neighboring property on interface.

**Results:**

We propose a novel statistical method to extract interacting residues, and interacting patches can be clustered as predicted interface residues. In addition, structural neighboring property can be adopted to construct a new energy function, for evaluating docking solutions. It includes new statistical property as well as existing energy items. Comparing to existing methods, our approach improves overall *F_nat _*value by at least 3%. On Benchmark v4.0, our method has average *I_rmsd _*value of 3.31*Å *and overall *F_nat _*value of 63%, which improves upon *I_rmsd _*of 3.89 *Å *and *F_nat _*of 49% for ZRANK, and *I_rmsd _*of 3.99*Å *and *F_nat _*of 46% for ClusPro. On the CAPRI targets, our method has average *I_rmsd _*value of 3.46 *Å *and overall *F_nat _*value of 45%, which improves upon *I_rmsd _*of 4.18 *Å *and *F_nat _*of 40% for ZRANK, and *I_rmsd _*of 5.12 *Å *and *F_nat _*of 32% for ClusPro.

**Conclusions:**

Experiments show that our method achieves better results than some state-of-the-art methods for identifying protein-protein binding sites, with the prediction quality improved in terms of CAPRI evaluation criteria.

## Introduction

The protein-protein interaction plays a key role in many biological functions, such as drug design and functional analysis. Gaining insights of various binding abilities will deepen our understanding on interaction. Determination of binding sites is widely applied in molecular biology research. Therefore, many efficient methods [1,2] have been developed for identifying binding sites.

Some existing approaches are based on analyzing differences between interface residues and non-interface residues, through machine learning methods or statistical methods. They analyze different features, such as sequence and structural properties or physical attributes. ProMate [3] creates interface or non-interface sphere around each residue. The histograms of many features are statistically obtained from spheres in training proteins. The probability for each sphere of a testing protein can be estimated to be on interface or not. The interface spheres are clustered to identify binding sites. PPI-Pred [4] uses several features to build an SVM model on interface prediction. It generates an interacting patch and a non-interacting patch for each training protein. Seven features are extracted from all interacting and noninteracting patches to predict if a testing patch is an interacting patch. Li *et al*. [5] divide protein residues into four different classes, which are distinguished by percentage of their neighboring interface residues. The core-SVM model is built over eight features and used to compute whether a residue is a core interface residue. In PINUP [6], an empirical scoring function consists of interface propensity and residue conservation score for predicting binding sites. PINUP takes top scoring patches and ranks residues based on their occurrences in these patches, clustered as predicted interface residues. Burgoyne *et al*. [7] analyze clefts on surface, that are likely to be binding sites. They can be ranked according to sequence conservation and physical properties. Meta-servers have also been constructed to combine strengths of some existing approaches. The program called meta-PPISP [8] combines three individual servers, namely cons-PPISP, ProMate and PINUP; another program called metaPPI [9] combines five prediction methods, namely PPI-Pred, PINUP, PPISP, ProMate, and Sppider.

In addition, several structural algorithms have also been used to identify binding sites, through analyzing surface structures. SiteEngine [10] recognizes surface regions of a testing protein that are similar to some known binding sites, using geometric hashing triangles. ProBiS [11] predicts interface residues by local surface structure alignment. It compares a testing protein to known binding sites, for detecting structurally similar residues. Ortuso *et al*. [12] define most relevant interaction areas, based on 3D maps. The GRID program is used to compute on known structural complexes.

Another kind of methods are to examine all possible poses of two protein subunits; that is, how subunits may dock. Docking methods based on fast Fourier transformation (FFT) [13], geometric surface matching [14], as well as intermolecular energy [15] have been proposed. ZRANK [16,17] combines an atom-based potential (IFACE) with five residue-based potentials for ranking docked conformations. It provides fast and accurate re-scoring of ZDOCK models [18]. ClusPro [19] develops a fast algorithm for filtering docked conformations with good surface complementarity, and ranks them based on their properties. RosettaDock [20] constructs an energy function using van der Waals energies, orientation-dependent hydrogen bonding, implicit Gaussian solvation, side-chain rotamer probabilities and a low-weighted electrostatics energy. HADDOCK [21] makes use of biochemical and biophysical interaction data, such as chemical shift perturbation data resulting from NMR titration experiments. Fernandez-Recio *et al*. [22] apply docking simulations and analyze interaction energy landscapes to identify interface residues. They use a global docking method based on multi-start energy optimization, and predict low-energy regions as binding sites.

Identifying of protein-protein interface depends on many features, such as sequence, structure, as well as other physicochemical properties. Hydrogen bonds and salt bridges are known to be essential in identifying binding specificity [23]. Most of binding sites are hydrophobic and conserved polar residues at specific locations [24]. Secondary structure composition analysis shows that neither helices nor *β*-sheets are dominantly populated on interface [25]. Several geometrical features such as weighted atomic packing density, relative surface area burial and weighted hydrophobicity are most effective features for predicting interface residues [26]. Some features only describe properties of current interacting residues, but cannot represent real situation well, thus are insufficient to predict binding sites with high accuracy.

In this paper, we analyze structural neighboring property on protein-protein interface, through Voronoi diagram. Using 6,438 complexes, we study local biases of structural neighboring property on interface. We propose a novel statistical method based on structural neighboring property to extract interacting residues, and interacting patches can be clustered as predicted interface residues. In addition, structural neighboring property can be adopted to limit the search space, for discovering native-like poses. Here, we construct an energy function to evaluate docking solutions, which includes new statistical property as well as existing energy items [27]. Finally, we use trained SVM models to further select best poses for each pair of input proteins.

Experiments show that our method achieves better results than some state-of-theart methods. Here, we use CAPRI evaluation criteria, *I_rmsd _*and *F_nat_*. Comparing to existing methods for identifying binding sites, our approach improves overall *F_nat _*value by at least 3%. On Benchmark v4.0, our method has average *I_rmsd _*value of 3.31*Å *and overall *F_nat _*value of 63%, which improves upon *I_rmsd _*of 3.89*Å *and *F_nat _*of 49% for ZRANK, and *I_rmsd _*of 3.99 *Å *and *F_nat _*of 46% for ClusPro. On CAPRI targets, our method has average *I_rmsd _*value of 3.46 *Å *and overall *F_nat _*value of 45%, which improves upon *Irmsd *of 4.18 *Å *and *F_nat _*of 40% for ZRANK, and *I_rmsd _*of *Å *and *F_nat _*of 32% for ClusPro.

## Methods

In this paper, we calculate structural neighboring property on protein-protein interface, through Voronoi diagram. We propose a novel statistical method to extract interacting residues, and interacting patches can be clustered as predicted interface residues. In addition, structural neighboring property can be adopted to construct an energy function to evaluate docking solutions, which includes new statistical property as well as existing energy items.

### Data set

To obtain statistical property on interface, we adopt a high quality, non-redundant experimental data set. We select 6,438 complexes from Protein Data Bank [28]; each complex consists of two or more subunits. These complexes are determined from X-ray data with resolution less than 2.2*Å*. Any two complexes share no more than 30% identity.

A complex may contain several subunits and multiple interfaces. Each interface in a complex occurs in a pair of subunits. Two residues between a pair of subunits are called interface residues, if any two atoms, one from each residue, interact. By interact, we mean distance between two heavy atoms is less than 6 *Å*.

### Structural neighboring property

Most of those features only describe current interacting residues, but cannot represent real situation well, thus are insufficient to predict binding sites with high accuracy. Here, we develop a method to calculate structural neighboring property on interface, using Voronoi diagram.

#### Site features

The physicochemical features are used to characterize potential interacting residues. The most interesting features are described as follows.

• Hydrophobicity: a numerical hydrophobicity of an amino acid [29],

• Electrostatic potential: the number of electrostatic charge in an amino acid [30],

• Hydrogen bonds: the number of potential hydrogen bonds for all atoms in an amino acid [31].

We use Voronoi diagram to evaluate polygonal face area of each surface residue, and calculate structural neighboring property on these physicochemical features.

#### Polygonal face area

We use VLDP [32] for geometrically analyzing protein 3D structures, based on Voronoi Tessellation. Voronoi Tessellation is a partition of space into polyhedra, whereas Delaunay diagram builds a graph with vertices at atoms. These graphs define nearest neighbours for each atom of one protein. It calculates Delaunay diagram by using an optimized incremental algorithm. The weights can be interpreted as squared radius. The surface residues appear as a packing of polyhedra, that is necessary to have a reasonable Tessellation throughout entire system.

VLDP can be used to evaluate residue contacts and residue volumes, defined as polygonal face area and polyhedral volume for each atom. In particular, *contact area *of two residues is sum of atomic interface areas on pairs of atoms; *surface area *of one residue is sum of surface areas exposed to solvent in this residue; *total area *of one residue is sum of all areas in this residue. We calculate structural neighboring property, based on polygonal face area.

#### Property function

**Figure 1:**
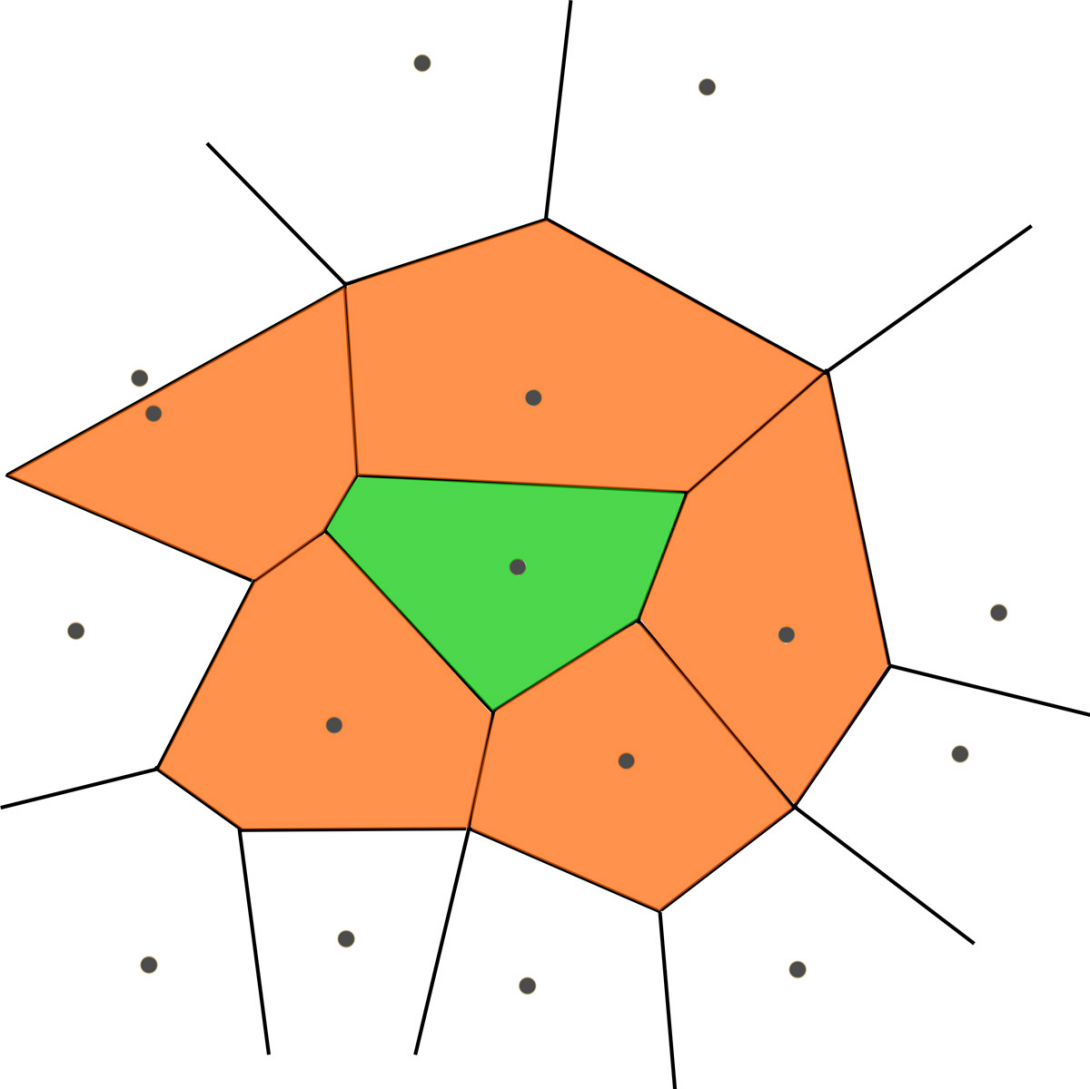
**Calculating structural neighboring property of residue *x *(green), based on neighboring residues *y *(orange)**.

Given a protein, structural neighboring property of one surface residue *x *is defined as follow:

$$p^{\prime }\left(x\right)=\frac{surface\left(x\right)}{total\left(x\right)}\times p\left(x\right)+\underset{contact\left(x;y\right)>0}{\sum }\frac{surface\left(y\right)}{total\left(y\right)}\times p\left(y\right)$$

where p(x) is site feature of each residue, *contact*(*x, y*) *>*0 means that contact area between residues *x *and *y *is greater than zero, $\frac{surface\left(x\right)}{total\left(x\right)}$ shows surface area of residue *x *divided by its total area, as shown in Figure 1.

We use a normal distribution *F *(*x*) to estimate probability of structural neighboring property on one side of interface. We also use a bivariate normal distribution *F *(*x*1*, x*2) [33] to estimate probability of structural neighboring property on both sides of interface. Given a pair of proteins, effective free energy of interacting residue pair can be calculated as:

$$S\left(x_{1},x_{2}\right)\;=-k_{B}T\underset{\left(x_{{}_{1}},x_{{}_{2}}\right)\in R}{\sum }\text{ln}\frac{F\left(x_{1},x_{2}\right)}{F\left(x_{1}\right)\;\times F\left(x_{2}\right)}$$

where *R *is a set of all residue pairs on interface.

### Extracting interface residues

We propose a statistical method to extract interacting residues, and interacting patches can be clustered as predicted interface residues. The threshold value *s_th _*is used to harvest all possible residue pairs between two proteins. The residue pairs with *S*(*x*_1_*, x*_2_) *≤ s_th _*are called interacting residues.

Considering neighboring residues, we construct a sphere with a radius of 10*Å *for all interacting residues. The updated statistical property of each interacting residue pair is calculated as follows.

$$\begin{array}{cc} S^{\prime }\left(x_{1},x_{2}\right)\text{ = }S\left(x_{1},\;x_{2}\right) & \text{ + }\underset{dis\left(x_{1},r_{i}\right)\leq 10Å}{\sum }\frac{1}{dis\left(x_{1},r_{i}\right)}S\left(r_{i},x_{2}\right)\\ & +\underset{dis\left(x_{2},r_{{}_{j}}\right)\leq 10Å}{\sum }\frac{1}{dis\left(x_{2},r_{{}_{j}}\right)}S\left(x_{1},r_{{}_{j}}\right) \end{array}$$

where *dis*(*x, r*) is 3D distance of two *C_α _*atoms in residues *x *and *r*. The residues *r_i _*are from protein having residue *x*_1_, and the residues *r_j _*are from protein having residue *x*_2_.

We rank interacting residues by using updated statistical property. Top interacting residues can be grouped into different regions. All interacting residues served as graph nodes, and each undirected edge is built when two nodes are within distance 10 *Å*. Strongly connected components are considered as interacting patches. One region, containing a very small number of interacting residues, indicates a weak signal and can be discarded. We cluster interacting patches as predicted interface residues.

### Energy function for docking

Given two input proteins, our task is to find the protein-protein interface between them. In first step, we identify docking solutions of two subunits. It performs a large number of rigid transformations to enumerate poses. Top ranking poses are selected through a linear combination of energy items. In second step, we generate possible conformational changes of interface residues from their unbound states to bound states, based on multidimensional scaling method. In third step, we use trained SVM models to further select best poses for input proteins. Structural neighboring property can be effectively applied to identify docking solutions.

Here, we construct a new energy function to evaluate docking solutions, which includes new statistical property as well as existing energy items [27]. The following lists all energy items, and how they are computed:

• Structural neighborhood energy is calculated by probability of structural neighboring property on interface.

• *π*-*π *interaction energy is calculated by geometrical property on *π*-*π *interaction [27].

• Dihedral angle energy is calculated by statistical analysis of dihedral angle frequency and correlation on interface [27].

• Amino acid energy is constructed by probabilities of interface residues.

• Side-chain atoms of interface residues are packed by SCWRL4 [34], and sidechain energy is extracted.

We use a linear combination of these energy items, referred to as initial energy function, to rank poses. The coefficient of each item is optimized by using a linear combination method in [35]. We output top 100 poses with lowest energy values. For conformational changed structures, our method calculates a set of possibly changed conformations of interfaces.

As in [27], we use a training set consisting of 79 complexes from Dockground [36] to produce 79 SVM models, one for each complex, based on these energy items. Finally, we use trained SVM models to further select best 10 poses with lowest energy values for two input proteins.

### Assessment of interface prediction

According to CAPRI evaluation criteria [37], three evaluation measures are commonly used in identifying protein-protein interface. A pair of residues on interface is considered to be in contact if any of their atoms are within 6 *Å *One is the fraction of native contacts *F_nat_*, defined as the number of correct residue-residue contacts in predicted complex divided by the number of contacts in native complex. The other is the fraction of non-native contacts *F_non−nat_*, defined as the number of incorrect residues-residue contacts in predicted complex divided by the total number of contacts in that predicted complex. The third is root-mean-square deviation of interface *I_rmsd_*, defined as the rmsd value between all backbone atoms of interfaces in predicted structure and in native complex, after two interfaces are superimposed.

We also calculate *P *value for binding sites prediction. The calculation of *P *value should be probability of obtaining not less than *n *correctly predicted interface residues by randomly picking out *N *predicted interface residues. The probability that a random method obtains success in one trial is$\frac{m}{M}$, where *M *is the number of all surface residues, and *m *is the number of correctly interface residues among them. Therefore, *P *value for binding sites prediction is given by

$$p\;=\sum_{i=n}^{N}\frac{N!}{n!\left(N-n\right)!}{\left(\frac{m}{M}\right)}^{n}{\left(1-\frac{m}{M}\right)}^{N-n}$$

## Results

In this section, we have done three kinds of experiments. First, we present statistical analysis of structural neighboring property on interface. Then, we compare our method to some existing methods, for identifying binding sites. The results show that our method performs better than other machine learning and statistical approaches. Finally, we examine docking solutions of our method on Benchmark v4.0 and CAPRI targets. Experiments show that our method outperforms some state-of-the-art methods.

### Statistical property

The physicochemical features are used to characterize potential interacting residues. The most interesting features are described by three values, as shown in Table 1.

**Table 1:** The physicochemical features of amino acids.

Amino Acid	Hydrophobicity	Electrostatic potential	Hydrogen bonds
Ala	1.8	0	2
Arg	-4.5	1	4
Asn	-3.5	0	4
Asp	-3.5	-1	4
Cys	2.5	0	2
Gln	-3.5	0	4
Glu	-3.5	-1	4
Gly	-0.4	0	2
His	-3.2	0	4
Ile	4.5	0	2
Leu	3.8	0	2
Lys	-3.9	1	2
Met	1.9	0	2
Phe	2.8	0	2
Pro	-1.6	0	2
Ser	-0.8	0	4
Thr	-0.7	0	4
Trp	0.9	0	3
Tyr	-1.3	0	3
Val	4.2	0	2

**Figure 2:**
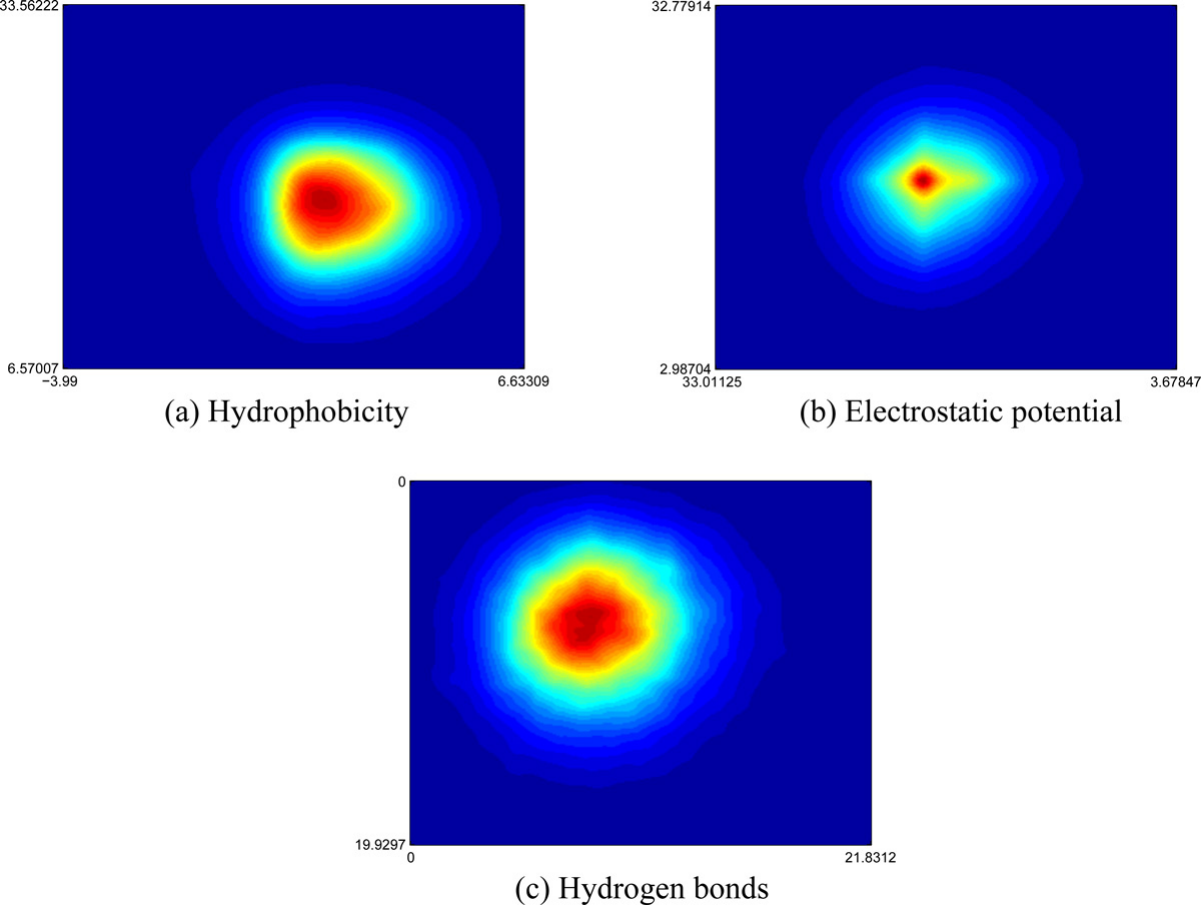
**The density plot of structural neighboring property on interface: (a) Hydrophobicity; (b) Electrostatic potential; (c) Hydrogen bonds**.

We present statistical analysis of structural neighboring property on interface. The statistics are carried out on 6,438 complexes. For each feature, we model a bivariate normal distribution. First, we represent an assessment for hydrophobicity on interface, as shown in Figure 2(a). The cluster centered at (1.89, 2.21) can be obtained. The groups of more hydrophobic amino acids often appear on interface. Second, local bias preferences of electrostatic potential on interface are shown in Figure 2b. We observe probability distribution centered at (0.12*, −*0.09). Many interfaces usually involve a lot of neutral amino acids. Third, we analyze hydrogen bonds on interface, as shown in Figure 2c. The data contains one cluster centered at (8.37, 7.96). The interface residues contain several potential hydrogen bonds, and the number of potential hydrogen bonds on each side of interface must be very similar.

We further investigate whether changing value of *s_th _*help to improve interface prediction. Here, we calculate structural neighboring property on 100 interface residues and 100 non-interface residues, randomly extracted from Benchmark v4.0 [38]. The relationship between interface prediction and the value of *s_th _*is shown in Table 2. We can observe that when increasing the value of *s_th_*, the overall *F_nat _*value of prediction is improved; however, the overall *F_non−nat _*value of prediction also increases as well.

**Table 2:** The relationship between interface prediction and the value of *s_th_*.

	*F_nat_*	*F_non−nat_*
*s_th _*= *−*100	38%	21%
*s_th _*= *−*50	62%	37%
*s_th _*= 0	65%	53%
*s_th _*= 50	68%	68%
*s_th _*= 100	69%	72%

### Binding sites prediction

Some existing methods use machine learning and statistical approaches to predict binding sites. The results show that our method performs better than other existing methods in binding sites prediction.

#### Comparison to Fernández-Recio's method

In this test, we compare the performance of our method to Fernández-Recio's method. The test data used by this method consists of 43 complexes [22]. The results are reported in Table 3. The overall *F_nat _*and *F_non−nat _*values for our method are 65% and 32%, respectively. Fernández-Recio method achieves overall *F_nat _*and *F_non−nat _*values of 62% and 60%, respectively.

**Table 3:** Comparison to Fernández-Recio method.

	Our method			Fernández-Recio		
		
	*F_nat_*	*F_non−nat_*	*P *value	*F_nat_*	*F_non−nat_*	*P *value
Overall	65%	32%	6.47E-6	62%	60%	0.002

#### Comparison to metaPPI, meta-PPISP and PPI-Pred

In this experiment, we compare our method to metaPPI, meta-PPISP and PPIPred. The test data consists of 41 complexes by metaPPI [9], divided into two categories: enzyme-inhibitor (EI) and others. The overall *F_nat _*and *F_non−nat _*values for each prediction method are reported in Table 4. The overall *F_nat _*values for our method, metaPPI, meta-PPISP and PPI-Pred achieve 62%, 28%, 38% and 38%, respectively. The overall *F_non−nat _*values for these four methods achieve 34%, 51%, 54% and 64%, respectively. Our method improves overall *F_nat _*value by at least 24%.

**Table 4:** Comparison to metaPPI, meta-PPISP and PPI-Pred.

Type	Our method			metaPPI			meta-PPISP			PPI-Pred		
				
	*F_nat_*	*F_non−nat_*	*P *value	*F_nat_*	*F_non−nat_*	*P *value	*F_nat_*	*F_non−nat_*	*P *value	*F_nat_*	*F_non−nat_*	*P *value
E-I^a^	65%	23%	1.15E-6	37%	39%	0.004	55%	44%	0.001	47%	54%	0.017
others	59%	42%	3.91E-4	22%	59%	0.128	26%	61%	0.137	31%	71%	0.206
Overall	62%	34%	3.09E-5	28%	51%	0.035	38%	54%	0.032	38%	64%	0.121

^a ^*E-I *is the type of enzyme-inhibitor.

#### Comparison to ProMate and PINUP

Our method is compared to ProMate and PINUP. The test data is originally used by ProMate [3], including 57 unbound proteins and their complexes. The results are reported in Table 5. The overall *_Fnat _*values for our method, PINUP and ProMate achieve 61%, 42% and 13%, respectively. The overall *F_non−nat _*values for these three methods achieve 45%, 55% and 47%, respectively. Our method improves overall *F_nat _*value by at least 19%.

**Table 5:** Comparison to PINUP and ProMate.

	Our method			PINUP			ProMate		
			
	*F_nat_*	*F_non−nat_*	*P *value	*F_nat_*	*F_non−nat_*	*P *value	*F_nat_*	*F_non−nat_*	*P *value
Overall	61%	45%	2.37E-4	42%	55%	0.025	13%	47%	0.161

#### Comparison to core-SVM

We compare our method to core-SVM with 50 dimers [5]. The results are reported in Table 6. The overall *F_nat _*values for our method and core-SVM are 63% and 60%, respectively. The overall *F_non−nat _*values for these two methods are 36% and 46%, respectively. Our method improves overall *F_nat _*value by at least 3%.

**Table 6:** Comparison to core-SVM.

	Our method			core-SVM		
		
	*F_nat_*	*F_non−nat_*	*P *value	*F_nat_*	*F_non−nat_*	*P *value
Overall	63%	36%	5.54E-5	60%	46%	2.42E-4

### Docking result

In this study, we compare our docking solutions with ZRANK [16,17] and external tool, FiberDock [39], specifically designed to handle conformation change after binding. We also compare our docking results with ClusPro [19]. For unbound-unbound docking, experiments show that our method significantly outperforms these existing docking approaches.

#### Training set

We consider 79 complexes from Dockground [36] as training set. In order to avoid over-fitting, we exclude complexes, which share more than 30 percent identity with cases in testing set. The average *I_rmsd _*value is 1.49*Å*, and the overall *F_nat _*and *F_non−nat _*values are 85% and 16%.

#### Evaluation on Benchmark v4.0

On Benchmark v4.0, the average *I_rmsd _*values predicted by our method, ZRANK+FiberDock and ClusPro are 3.31*Å*, 3.89*Å *and 3.99*Å*, respectively. The overall *F_nat _*values predicted by these three methods are 63%, 49% and 46%, respectively. The results are shown in Table 7.

**Table 7:** The prediction results by our method, ZRANK+FiberDock and ClusPro on Benchmark v4.0.

Subset^a^	No. of cases	our method			ZRANK+FiberDock			ClusPro		
				
		*I_rmsd_*	*F_nat_*	*F_non−nat_*	*I_rmsd_*	*F_nat_*	*F_non−nat_*	*I_rmsd_*	*F_nat_*	*F_non−nat_*
Rigid body	123	2.89	69%	35%	3.31	56%	49%	3.33	55%	51%
Medium difficult	29	3.38	59%	39%	4.46	39%	59%	4.71	30%	69%
Difficult	24	5.41	36%	58%	6.18	28%	67%	6.53	21%	77%
Overall	176	3.31	63%	39%	3.89	49%	53%	3.99	46%	58%

^a ^Subset is based on the magnitude of conformational change after binding.

The complexes are classified into three categories, according to the magnitude of conformational change after binding. In rigid-body group, the average *I_rmsd _*values predicted by our method, ZRANK and ClusPro are 2.89*Å*, 3.31*Å *and 3.33*Å*, respectively. The overall *F_nat _*values predicted by these three methods are 69%, 56% and 55%, respectively. In medium difficulty group, the average *I_rmsd _*values predicted by our method, ZRANK+FiberDock and ClusPro are 3.38*Å*, 4.46*Å *and 4.71*Å*, respectively. The overall *F_nat _*values predicted by these three methods are 59%, 39% and 30%, respectively. In difficulty group, the average *I_rmsd _*values predicted by our method, ZRANK+FiberDock and ClusPro are 5.41*Å*, 6.18*Å *and 6.53*Å*, respectively. The overall *F_nat _*values predicted by these three methods are 36%, 28% and 21%, respectively.

#### Evaluation on CAPRI

We evaluate docking solutions of our method, ZRANK and ClusPro on CAPRI targets. CAPRI [37] is a community-wide experiment to assess the capacity of docking methods. The average *I_rmsd _*values predicted by our method, ZRANK+FiberDock and ClusPro are 3.46*Å*, 4.18*Å *and 5.12*Å*, respectively. The overall *Fnat *values predicted by these three methods are 45%, 40% and 32%, respectively. The results are shown in Table 8.

**Table 8:** The prediction results by our method, ZRANK+FiberDock and ClusPro on CAPRI targets.

Target	our method			ZRANK+FiberDock			ClusPro		
			
	*I_rmsd_*	*F_nat_*	*F_non−nat_*	*I_rmsd_*	*F_nat_*	*F_non−nat_*	*I_rmsd_*	*F_nat_*	*F_non−nat_*
T01	4.54	10%	89%	8.10	7 %	88%	12.6	0 %	100%
T02	1.53	87%	11%	0.51	96%	3 %	19.0	0 %	100%
T03	7.69	8 %	91%	1.92	60%	37%	3.61	23%	67%
T04	3.98	34%	60%	4.56	23%	72%	10.5	1 %	85%
T05	9.74	7 %	72%	10.1	5 %	90%	1.95	56%	38%
T06	5.76	16%	66%	3.10	28%	70%	3.68	23%	69%
T07	4.77	11%	87%	6.43	3 %	88%	12.1	0 %	100%
T08	6.07	16%	69%	1.09	47%	51%	6.50	8 %	91%
T09	2.85	33%	66%	9.77	8 %	80%	24.7	0 %	100%
T10	3.52	29%	66%	5.05	11%	77%	6.18	5 %	88%
T11	2.56	61%	35%	2.63	61%	38%	3.12	42%	54%
T12	1.55	76%	23%	0.65	84%	15%	0.78	93%	4 %
T13	0.63	94%	4 %	2.38	54%	39%	3.98	32%	64%
T14	9.62	4 %	87%	0.95	73%	25%	1.89	51%	45%
T15	1.40	69%	28%	0.86	91%	7 %	1.83	51%	47%
T18	3.08	25%	67%	1.86	66%	31%	3.70	21%	69%
T19	1.74	59%	38%	10.3	3 %	88%	2.58	32%	62%
T20	7.48	5 %	83%	6.31	7 %	79%	3.24	21%	74%
T21	1.56	84%	15%	3.23	36%	59%	2.78	67%	32%
T22	2.48	75%	19%	5.61	5 %	86%	3.12	42%	49%
T23	1.90	61%	34%	1.34	72%	27%	4.80	16%	70%
T24	2.01	50%	48%	3.13	20%	75%	5.65	2 %	89%
T25	2.13	57%	40%	1.51	64%	33%	1.85	65%	32%
T26	0.89	84%	14%	0.93	78%	20%	1.21	54%	43%
T27	1.95	60%	39%	1.86	59%	37%	3.70	21%	73%
T29	2.46	69%	25%	3.13	49%	50%	3.57	42%	49%
T30	7.48	9 %	79%	4.84	16%	77%	5.40	11%	75%
T32	2.98	34%	59%	9.45	3 %	95%	0.52	87%	12%
T35	3.71	29%	62%	8.71	4 %	82%	6.90	7 %	83%
T36	3.70	27%	69%	3.64	25%	66%	6.20	9 %	79%
T37	1.25	53%	45%	0.93	92%	7 %	6.89	5 %	88%
T39	0.87	75%	24%	15.6	0 %	100%	1.60	56%	42%
T40	2.17	56%	43%	0.43	86%	13%	1.17	62%	36%
T41	1.09	67%	30%	1.45	46%	51%	1.20	51%	48%
T42	3.70	28%	68%	4.13	15%	75%	0.91	75%	24%

### Assessment of energy items

To assess effectiveness of energy items, we re-optimize coefficients in each case with only four of five items. We evaluate docking poses of 176 complexes on Benchmark v4.0, by leaving one energy item out. The results are shown in Table 9. The overall *F_nat _*value for case without *E_nb _*is 58.6%, for case without *E_pi _*is 60.5%, for case without *E_da _*is 60.2%, for case without *E_aa _*is 59.3%, and for case without *E_sc _*is 58.1%. The average *I_rmsd _*values of five cases are less than that for case with all items. As can be seen, five energy items are all effective, among which structural neighborhood energy item is the most effective one.

**Table 9:** Performance of different energy items on Benchmark v4.0.

	*I_rmsd_*	*F_nat_*
Case without *E_nb_*	3.48	58.6%
Case without *E_pi_*	3.39	60.5%
Case without *E_da_*	3.37	60.2%
Case without *E_aa_*	3.45	59.3%
Case without *E_sc_*	3.52	58.1%
Case with all items	3.31	63.0%

## Conclusion

In this paper, we calculate structural neighboring property on interface, through Voronoi diagram. We propose a novel statistical method to extract interacting residues, and interacting patches can be clustered as predicted interface residues. Experiments show that our method achieves better results than some state-of-the-art methods. Comparing to existing methods for binding sites prediction, our approach improves overall *F_nat _*value by at least 3%.

In addition, structural neighboring property can be adopted to construct an energy function, for evaluating docking solutions. It includes new statistical property as well as existing energy items. On Benchmark v4.0, our method has average *I_rmsd _*value of 3.31*Å *and overall *F_nat _*value of 63%. On CAPRI targets, our method has average *I_rmsd _*value of 3.46*Å *and overall *F_nat _*value of 45%.

### Availability

The test sets of protein complexes and the prediction results are available here

https://sites.google.com/site/guofeics/structural_neighboring_property.

## Competing interests

The authors declare that they have no competing interests.

## Authors' contributions

FG and LW conceived the study. FG and ZW performed the experiments and analyzed the data. FG and LW drafted the manuscript. All authors read and approved the manuscript.

## Declarations

Funding for publication of this article comes from National Science Foundation of China [NSFC 61402326, 61373048].

This article has been published as part of *BMC Systems Biology *Volume 9 Supplement 5, 2015: Selected articles from the IEE International Conference on Bioinformatics and Biomedicine (BIBM 2014): Bioinformatics. The full contents of the supplement are available online at http://www.biomedcentral.com/bmcsystbiol/supplements/9/S5.
